# A multiscale modeling study of particle size effects on the tissue penetration efficacy of drug-delivery nanoparticles

**DOI:** 10.1186/s12918-017-0491-4

**Published:** 2017-11-25

**Authors:** Mohammad Aminul Islam, Sutapa Barua, Dipak Barua

**Affiliations:** Department of Chemical and Biochemical Engineering, University of Missouri Science and Technology, Rolla, Missouri USA

**Keywords:** Brownian dynamics, Tumor, Drug delivery, Porous media, Diffusion

## Abstract

**Background:**

Particle size is a key parameter for drug-delivery nanoparticle design. It is believed that the size of a nanoparticle may have important effects on its ability to overcome the transport barriers in biological tissues. Nonetheless, such effects remain poorly understood. Using a multiscale model, this work investigates particle size effects on the tissue distribution and penetration efficacy of drug-delivery nanoparticles.

**Results:**

We have developed a multiscale spatiotemporal model of nanoparticle transport in biological tissues. The model implements a time-adaptive Brownian Dynamics algorithm that links microscale particle-cell interactions and adhesion dynamics to tissue-scale particle dispersion and penetration. The model accounts for the advection, diffusion, and cellular uptakes of particles. Using the model, we have analyzed how particle size affects the intra-tissue dispersion and penetration of drug delivery nanoparticles. We focused on two published experimental works that investigated particle size effects in in vitro and in vivo tissue conditions. By analyzing experimental data reported in these two studies, we show that particle size effects may appear pronounced in an in vitro cell-free tissue system, such as collagen matrix. In an in vivo tissue system, the effects of particle size could be relatively modest. We provide a detailed analysis on how particle-cell interactions may determine distribution and penetration of nanoparticles in a biological tissue.

**Conclusion:**

Our work suggests that the size of a nanoparticle may play a less significant role in its ability to overcome the intra-tissue transport barriers. We show that experiments involving cell-free tissue systems may yield misleading observations of particle size effects due to the absence of advective transport and particle-cell interactions.

**Electronic supplementary material:**

The online version of this article (doi:10.1186/s12918-017-0491-4) contains supplementary material, which is available to authorized users.

## Background

Drug-delivery nanoparticles are subject to a variety of transport barriers in biological tissues [1, 2]. To overcome these barriers, significant research efforts have been made over the years to study the principles of drug-delivery nanoparticle design [3]. The key nanoparticle design features that have been widely studied are particle size, geometry, and surface-attached targeting molecules [4]. Among these, the size of a particle is believed to have important effects on its immune clearance, transvascular delivery, and intra-tissue dispersion and penetration [4, 5].

Two earlier studies quantitatively studied the effects of particle size on the efficacy of tissue delivery and penetration of drug-delivery nanoparticles [6, 7]. Nonetheless, the mechanistic aspects of these effects remain poorly understood. Earlier, an experiment by Wong et al. [6] indicated enhanced tissue penetration as a result of particle size reduction. Later, Tang et al. [7] reported similar effects from particle size variation but their experimental data revealed significantly narrower tissue distribution profiles and penetration of particles. Moreover, in Tang et al. [7], the effects of particle size variation appeared relatively modest. These apparent disparities motivated us to develop a multiscale model and mechanistically interrogate particle size effects on their efficacy of tissue distribution and penetration. The two studies above carried out investigations in different experimental settings. Wong et al. [6] employed in vitro experiments involving a cell-free collagen tissue. On the other hand, the experiments of Tang et al. [7] were conducted in in vivo tumor tissues. We were particularly interested in investigating how these two experimental settings might affect the intra-tissue transport behavior and penetration efficacy of nanoparticles of different sizes.

We developed the multiscale model to realistically capture the transport behavior and cellular interactions of nanoparticles. In many aspects, a biological tissue can be compared with a heterogeneous porous media. Particle motion through the interstitial space of a biological tissue is subject to advection, diffusion, and interaction with the cell boundaries. Tissue-scale particle distribution may occur over hours. However, the process is ultimately determined by the micro scale adhesion and interaction of particles with the cell boundaries. Bridging these spatiotemporal phenomena at distinct spatial and temporal resolutions in a model could be computationally expensive. Here, we developed a time-adaptive Brownian Dynamics (BD) simulation algorithm. We combined the algorithm with the Method of Regularized Stokeslets (MRS) [8]. The integrated algorithm enabled multiscale simulation of particle transport under both advection and diffusion in a heterogeneous porous system. The time-adaptive feature captured particle-cell interactions at high resolution while enabling efficient computation.

Using the model, we analyzed experimental data reported in Wong et al. [6] and Tang et al. [7]. Our analysis revealed how the different tissue conditions in these two experimental studies could lead to the distinct particle distribution profiles and size effects. Our results and analysis indicate that particle size effects may appear pronounced in a cell-free tissue system, such as collagen matrix, often employed in in vitro microfluidic studies. In the absence of particle-cell interaction and under pure diffusion, particle size may have more dramatic effects on the tissue distribution and penetration efficacy of nanoparticles. However, in in vivo physiological conditions, the barriers imposed by the interstitial cells may moderate the effects arising from the particle size difference. We show that particle-cell interaction imposes significant transport barriers and serves as a key determinant of distribution and penetration efficacy of nanoparticles.

## Methods

Below, we describe our simulation approach together with the model of nanoparticle transport in biological tissues. The model is written in C++. The source code for the model and associated instructions are available in Additional file 1 (S1_File.zip).

### Domain representation of biological tissue

**Fig. 1:**
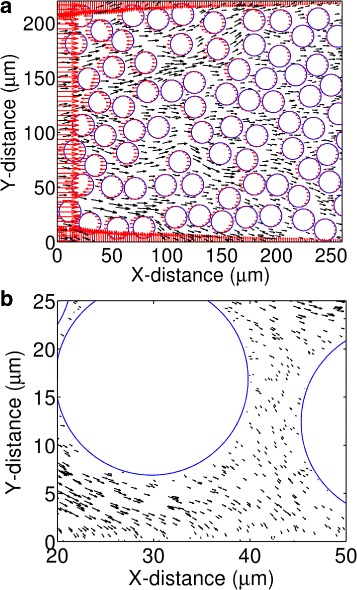
The MRS calculated force and velocity fields in a rectangular tissue section. **a** The red arrows represent force vectors at discrete locations along the domain edges and cell boundaries. The black arrows represent velocity vectors in the interstitial space. **b** A zoomed-in view of the velocity vectors in the interstitial space and near the cell boundaries

The computational domain in our model represents a two-dimensional rectangular tissue section (Fig. 1 a). We refer the entire domain by *Ω*, and its left, lower, and upper edges by *Ω*_1_, *Ω*_2_, and *Ω*_3_, respectively. We consider the rectangle sufficiently wide such that the right edge can be ignored. The bottom-left corner of the domain (*Ω*_1_∩*Ω*_2_) represents the origin, and any point ***x***∈*Ω* represents a position with respect to this origin. The left edge, *Ω*_1_, represents a porous capillary wall from where nanoparticles enter into the tissue space. The entry points of particles, ***x***∈*Ω*_1_, are selected randomly along this edge. The horizontal distance to the right with respect to *Ω*_1_ represents tissue depth (labeled as X-distance in Fig. 1).

We treat the mobile nanoparticles as circular objects with a defined size (radius). We treat each cell as a stationary circle of 10 *μ*m radius. Cells are populated at non-overlapping random positions in the domain. The cells occupy 40% area of the domain. We refer this aggregate area occupied by the cells as *Λ*. The remaining 60% area represents the interstitial space, which we refer to as *Γ*. We refer the boundary of any cell *i*∈{1,2,⋯,*n*} as *P*_*i*_, and the region the cell occupies as *A*_*i*_. Therefore $\Lambda = (\cup _{i=1}^{n}P_{i}) \cup (\cup _{i=1}^{n}A_{i})$. Thus, the entire computational domain, *Ω* is equal to $(\cup _{i=1}^{3}\Omega _{i})\cup \Gamma \cup \Lambda $.

### Nanoparticle velocity

To evaluate nanoparticle velocities in the domain, we adopt the approach of Rejniak et al. [9]. At any position ***x***∈*Ω*, we represent the velocity of a nanoparticle by the local fluid velocity ***v(x)*** [9]. As in [9], we compute ***v(x)*** using the Method of Regularized Stokeslets (MRS) [8]. The MRS [8] has been used to model complex solid-fluid interactions in a variety of Stokes flow systems [10–14]. Here, for completeness, we provide a brief description of the MRS and its implementation in our model.

#### The method of regularized stokeslets (MRS)

The MRS is a Lagrangian approximation of the Stokes equations. It provides a convenient framework to avoid singularities associated with the fundamental solutions of the Stokes equations. Because of this property, the method is particularly useful for modeling Stokes flow associated with irregular geometries or non-smooth boundaries.

The Stokes equations in two or three dimension are as follows:

$$\begin{array}{*{20}l} &\mu\nabla \boldsymbol{u}(\boldsymbol{x}) = \nabla P - \boldsymbol{f} \\ &\nabla\cdot\boldsymbol{u}(\boldsymbol{x})=0 \end{array} $$

In the above equations, *μ* is the fluid viscosity; ***x*** is a position vector; ***f*** is force; and *P* is pressure. ***u***(***x***) is the local fluid velocity vector at ***x***. The Stokes equations can be solved for a single point force at ***x***_0_, ***f***=***f***_0_ *δ*(***x***−***x***_0_), where *δ*(***x***) represents the Dirac delta function.

$$\begin{array}{*{20}l} &\mu\nabla \boldsymbol{u}(\boldsymbol{x}) = \nabla p(\boldsymbol{x}) - \boldsymbol{f}_{0}\delta(\boldsymbol{x}-\boldsymbol{x}_0) \\ &\nabla\cdot\boldsymbol{u}(\boldsymbol{x})=0 \end{array} $$

The solution of the above equations represents the velocity ***u***(***x***) at ***x*** due to the single point force at ***x***_0_. This solution, however, is singular at the point of application of the force (i.e., |***u***(***x***)|→*∞*, as ***x***→***x***_0_). To avoid this singularity, the MRS avoids direct use of the point force ***f***_0_ *δ*(***x***−***x***_0_) in the Stokes equations. Instead, it approximates (regularizes) the point force into a smooth, radially-symmetric force centered at ***x***_0_: ***f***_0_ *ϕ*_*ε*_(***x***−***x***_0_). With this regularized force term, the Stokes equations take the following form:

1 $$\begin{array}{*{20}l} \mu\nabla \boldsymbol{u}(\boldsymbol{x}) = \nabla P(\boldsymbol{x}) - \boldsymbol{f}_{0}\phi_{\epsilon}(\boldsymbol{x}-\boldsymbol{x}_0) \end{array} $$

2 $$\begin{array}{*{20}l} \nabla\cdot\boldsymbol{u}(\boldsymbol{x})=0 \end{array} $$

The function *ϕ*_*ε*_(***x***) is known as cutoff function, which represents a spatially-symmetric sphere or blob of radius *ε* in the domain space. The regularized force ***f***_0_ *ϕ*_*ε*_(***x***−***x***_0_) takes the maximum value at the center (***x***_0_), and decays smoothly towards the surface of the blob. The cutoff function satisfies the constraint $\int _{-\infty }^{+\infty } \phi _{\epsilon }(\boldsymbol {x})d(\boldsymbol {x}) = 1$. As *ε*→0, *ϕ*_*ε*_(***x***)→*δ*(***x***), and the regularized force approaches the point force.

For an appropriate choice of the cutoff function *ϕ*_*ε*_(***x***), Eqs. 1 and 2 can be solved to evaluate the fluid velocity ***u***(***x***) due to the regularized point force centered at any arbitrary position ***x***_0_ in the fluid. Unlike the Stokes solution, the resulting velocity is non-singular at ***x***_0_.

Now, the force field over the entire domain can be represented by a collection of *N* discrete point forces located at different points in the domain. If ***f***_*k*_ located at ***x***_*k*_ for *k*∈{1,2,⋯,*N*} represents such a point force, its contribution at ***x*** can be represented as ***u***_*k*_(***x***). By solving Eqs. 1 and 2, ***u***_*k*_(***x***) for *k*∈{1,2,⋯,*N*} can be evaluated. Then, the net velocity at ***x***, ***v***(***x***), can be evaluated simply by linear superposition of the solutions corresponding to the *N* discrete forces: $\boldsymbol {v}(\boldsymbol {x})=\sum _{k=1}^{N} \boldsymbol {u}_{k}(\boldsymbol {x})$

#### Force and velocity calculation

Following Rejniak et al. [9] and Tlupova et al. [15], we chose $\phi _{\epsilon }(\boldsymbol {x}) = \frac {2\epsilon ^{4}}{\pi (r^{2}+\epsilon ^{2})^{3}}$, where *r*=|***x***|. We discretized the solid boundaries of the tissue domain into *N*=6,700 discrete points. The solid boundaries include the three domain edges (*Ω*_1_, *Ω*_2_, and *Ω*_3_), and the boundaries of the circular cells, *P*_*i*_ for *i*∈{1,2,⋯,*n*}.

For the above cutoff function, the solution of Eqs. 1 and 2 is:

3 $$\begin{array}{@{}rcl@{}} \boldsymbol{u}_{k}(\boldsymbol{x})&=&-\frac{\boldsymbol{f}_{k}}{8\pi\mu}\left(\ln \big(r^{2} +\epsilon^{2}\big) - \frac{2\epsilon^{2}}{r^{2}+\epsilon^{2}}\right)  \\ && + \frac{1}{4\pi\mu} \frac{1}{r^{2}+\epsilon^{2}}\left[~\boldsymbol{f}_{k}.\left(\boldsymbol{x}-\boldsymbol{x}_{k}\right)\right]\left(\boldsymbol{x}-\boldsymbol{x}_{k}\right)~.  \end{array} $$

For the entire collection of the *N* discrete forces, the net velocity ***v***(*x*) is obtained by linear addition of the solutions:

4 $$\begin{array}{@{}rcl@{}} \boldsymbol{v}(\boldsymbol{x})&=&\sum\limits_{k=1}^{N}{\boldsymbol{u}_{k}(\boldsymbol{x})}  \\ &=&\sum_{k=1}^{N} \left\{-\frac{\boldsymbol{f}_{k}}{8\pi\mu}\left(\ln \left(r^{2} +\epsilon^{2}\right) - \frac{2\epsilon^{2}}{r^{2}+\epsilon^{2}}\right)\right.  \\ &&\left. + \frac{1}{4\pi\mu} \frac{1}{r^{2}+\epsilon^{2}}\left[~\boldsymbol{f}_{k}.\left(\boldsymbol{x}-\boldsymbol{x}_{k}\right)\right]\left(\boldsymbol{x}-\boldsymbol{x}_{k}\right)\right\}~.  \end{array} $$

However, to obtain ***v***(***x***) using Eq. 4 (or ***u***_*k*_(***x***) using Eq. 3), we had to first evaluate the unknown point forces, ***f***_*k*_s, at the *N* discrete points. To evaluate the ***f***_*k*_s, we set no-slip boundary conditions (***u***_*k*_=0) at the lower and upper domain edges (*Ω*_2_ and *Ω*_3_), and the cell boundaries *P*_*i*_ for *i*∈{1,2,⋯,*n*}. As mentioned previously, the left domain edge *Ω*_1_ represents the particle or fluid entry points (the porous wall of a vascular capillary). At *Ω*_1_, we set the boundary condition $\boldsymbol {u}_{k} = 1\boldsymbol {\hat {j}}$ *μ*m/second, where $\boldsymbol {\hat {j}}$ represents a unit vector towards the tissue depth (parallel to *Ω*_2_ or *Ω*_3_). Thus, for the *N* discrete points, we obtained a system of *N* independent linear equations from Eq. 4. The left hand-side (***u***(***x***)) of these equations were defined (either 0 or $\boldsymbol {\hat {j}}$), whereas the right-hand side contained the *N* unknown force terms ***f***_*k*_s. Using the GSL package (ftp://ftp.gnu.org/gnu/gsl/), we solved this system of linear equations to evaluate the unknown ***f***_*k*_s at the *N* discrete points. We then plugged these force terms into Eq. 4 to evaluate the velocity vector ***v***(***x***) at any arbitrary position ***x*** in the interstitial space of the domain.

In Fig. 1, we represent the force vectors, ***f***_*k*_s by red arrows. The length and direction of each red arrow represent the relative magnitude and direction of the corresponding force vector at the indicated point. We represent the velocity vectors at different points of the interstitial space by black arrows. The length and direction of each black arrow represent the relative magnitude and direction of the fluid (nanoparticle) velocity at the indicated point.

### Nanoparticle diffusion

We calculated diffusion constants of the nanoparticles based on the Einstein-Stokes equation:

5 $$ D = \frac{K_{B}T}{6\pi\mu a}  $$

where *D* is diffusion constant of a particle, *K*_*B*_ is the Boltzmann constant, *T* is temperature, *μ* is viscosity of the interstitial fluid, and *a* is radius of the particle.

### Time-adaptive simulation algorithm

In our BD algorithm, we consider that the nanoparticles are independent and mutually non-interacting in a biological tissue. This consideration is based on the fact that drug-delivery nanoparticles can reach a target tissue at small quantities. Typical particle concentration in a biological tissue is expected to be small. Therefore, it is less likely that their mutual interaction can have a significant impact on their transport behavior over other factors, such as fluid flow, collision with the cell boundaries, and cellular uptake. Because particles are considered independent, the model allows independent simulation of one particle at a time.

**Fig. 2:**
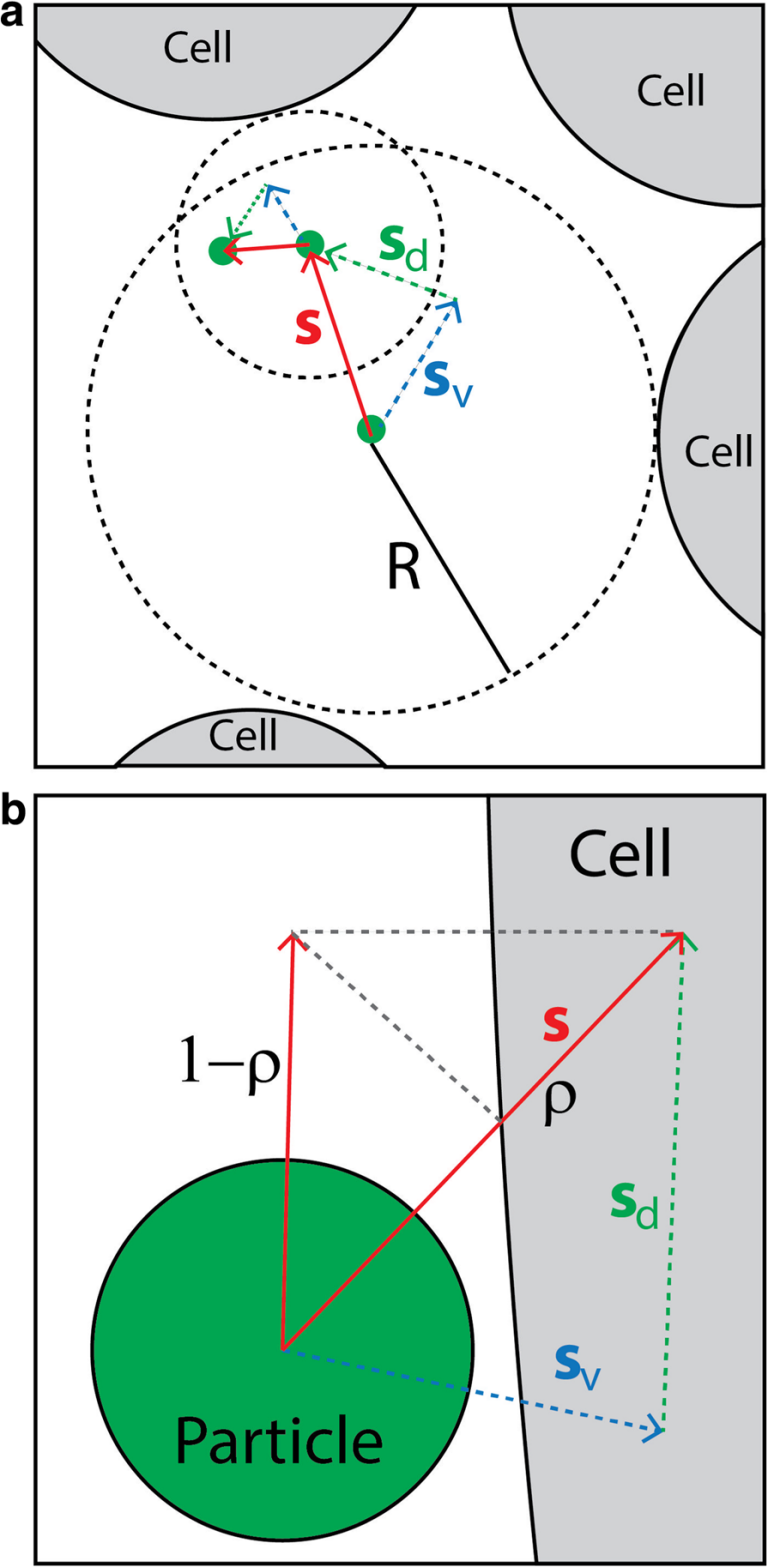
Illustration of the time-adaptive BD algorithm. **a** Particle motion in the bulk fluid. The small green circle represents a nanoparticle, and the large gray circles represent cells. The radius of the dashed circle, *R*, represents the distance between a particle’s current position and its nearest cell boundary. In the bulk fluid, particle jump ***S*** is taken adaptively so that |***S***|<*R*−*a*. |***S***| is determined by the time step *Δ* *t*: ***S***=***S***_*v*_+***S***_***d***_, where ***S***_*v*_=***v*** *Δ* *t* (displacement due to advection), and $\boldsymbol {S}_{d} = \sqrt {4D\Delta t}\boldsymbol {e}$ (displacement due to diffusion). **b** Particle motion near a cell boundary. |***S***| is determined by a constant but fine resolution time step *δ* *t*=10^−3^ seconds. The cell boundary represents a sticky wall that captures or reflects a colliding particle with probability *ρ* and 1−*ρ*, respectively

**Fig. 3:**
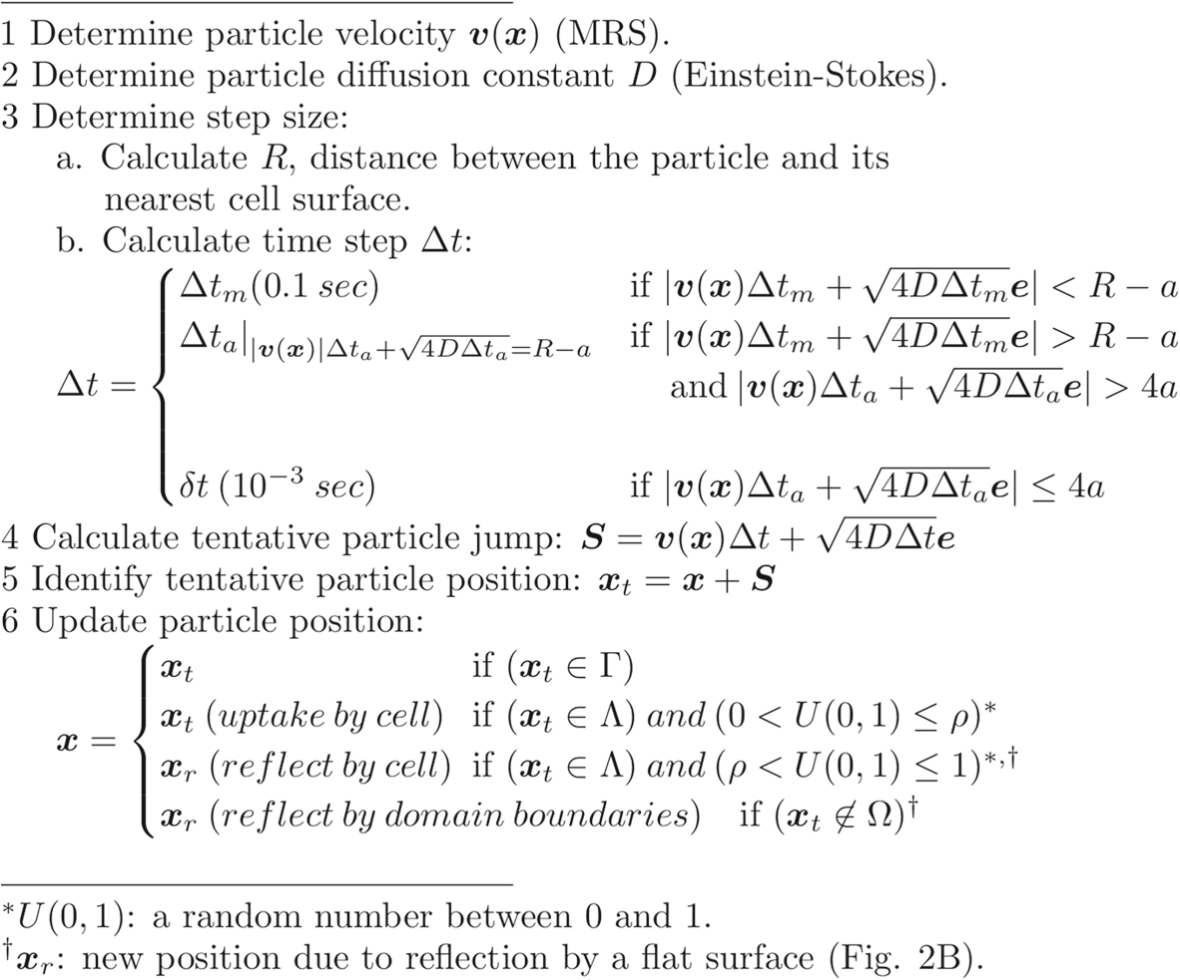
Pseudocode for the simulation algorithm

Figure 2 illustrates the time-adaptive scheme of the algorithm. The algorithm is summarized in a pseudocode in Fig. 3. In the algorithm, particles are advanced adaptively with time steps *Δ* *t*_*m*_≥*Δ* *t*≥*δ* *t*, where *Δ* *t*_*m*_ and *δ* *t* represent the largest and smallest permissible time step, respectively.

During the simulation, in each BD step, the algorithm first computes *R*, which is the distance between the center of a particle and its nearest interaction point on a solid boundary (Fig. 2). The solid boundary can be any of the three domain edges or cell boundaries. It then attempts to move the particle based on the largest permissible step *Δ* *t*_*m*_. It computes a possible jump: $\boldsymbol {S} = \boldsymbol {S}_{v} + \sqrt {4D\Delta t_{m}}\boldsymbol {e}$, where ***S***_*v*_=***v*** *Δ* *t*_*m*_ represents displacement due to advection, $\boldsymbol {S}_{d} = \sqrt {4D\Delta t_{m}}$ represents displacement due to diffusion (Fig. 2), and ***e*** represents a unit vector with random orientation. Velocity ***v*** and diffusion constant *D* are computed using the MRS and Einstein-Stokes equation, as detailed in the previous sections. If the jump length |***S***| is smaller than *R*−*a*, where *a* is the particle radius, the move is accepted, and the particle position is updated accordingly.

If the move based on *Δ* *t*_*m*_ is rejected, the algorithm attempts to move the particle based on a new time step *Δ* *t*_*a*_<*Δ* *t*_*m*_. This time step *Δ* *t*_*a*_ is obtained by solving |***v***|*Δ* *t*_*a*_ + $\sqrt {4D\Delta t_{a}} = R-a$. It then computes: $\boldsymbol {S} = \boldsymbol {v}\Delta t_{a} + \sqrt {4D\Delta t_{a}}\boldsymbol {e}$. The algorithm then compares |***S***| with the particle radius *a*. If |***S***|>4*a* (i.e., the distance between the particle boundary and a cell boundary is at least twice the diameter of the particle), the move is accepted and the particle position is updated accordingly.

If |***S***|≤4*a*, the algorithm attempts to move the particle based on the smallest permissible step *δ* *t*: $\boldsymbol {S} = \boldsymbol {v}\delta t + \sqrt {4D\delta t}\boldsymbol {e}$. The move is accepted if the new particle position falls in the interstitial space (*Γ*). However, if the new position falls outside the domain edges, or in any of the cell regions (*Λ*), the algorithm treats it as a collision with the corresponding domain edge or cell boundary. In the former case, the particle is reflected by the domain boundary. In the latter case, the particle is captured or reflected by a cell boundary, as discussed in the next section.

### Particle interaction with cell boundaries

We consider the cell boundaries as sticky walls that can capture or reflect a hitting nanoparticle with a defined probability (Fig. 2 b). Because a cell is much larger in size than a particle, a cell boundary is treated as a flat surface when a particle collides with the boundary (Fig. 2 b). As mentioned in the previous section, a particle can hit a cell only when it is in the vicinity of a cell and advanced by the finest time step *δ* *t*=10^−3^ s (Additional file 2: A and B). This time step size requires the distance between a colliding particle and a cell boundary to be small (four times the particle radius). When a particle hits a cell, it is either captured with probability *ρ*, or reflected into the fluid with probability (1−*ρ*) (Fig. 2 b). The value of *ρ* determines the rate of particle capture (uptake) by cells.

It should be noted that particle capture or uptake by a cell may involve complex biophysical and biochemical processes. These processes can be influenced by many factors, such as van der Waals force [16], particle surface charge effects [17], particle surface modification by corona formation [18–21], and molecular recognition by the receptor proteins in the cell membrane [22–25]. Explicit consideration of these different factors may be possible if quantitative information about their relative importance and molecular mechanisms of the recognition processes are known. Here, we limit our scope by taking this simple approach where the probability parameter *ρ* implicitly accounts for the lumped effects from the various factors that may influence particle capture by cells. For example, a particle with a small *ρ* in our model may represent a particle with a bare surface with a poor affinity for the cell membrane. On the other hand, a particle with a large *ρ* may represent a particle with a modified surface (functionalized with a targeting ligand, for example) with a high affinity for the cell membrane because of the molecular recognition by membrane proteins [17, 22–25].

### Model parameters

Table 1 lists the model parameters and their values. In the model, cells have a typical radius of 10 *μ*m. Nanoparticles have a radius of 100 nm if a different size is not specified explicitly. Tissue porosity (*Γ*/*Ω*) is 0.60. The probability of particle capture per collision with a cell (*ρ*) is varied between 0.01 and 1. Physiological temperature (37°C or 310 K) was used in the Einstein-Stokes equation to calculate particle diffusion. The remaining parameters, fluid viscosity (*μ*), entry fluid velocity (*v*_*in*_), and regularization constant (*ε*) are based on [9]

**Table 1:** Model parameter values

Parameter	Value	Reference
Cell radius, *r* (*μ*m)	10	This work
Nanoparticle radius, *a* (nm)	10– ***100*** ^∗^	This work
Tissue porosity, *α*	0.6	This work
Particle capture probability, *ρ*	^∗^ ***0.01***–1	This work
Fluid viscosity, *μ* (cP)	2.5	[9]
Temperature, *T* (K)	310	
Entry fluid velocity, *v*_*in*_ (*μ*m/s)	0.05–1	[9]
Regularization constant, *ε* (*μ*m)	0.5	[9]

^*^Bold font indicates the default parameter value used in the simulations

## Results

### Size effects of nanoparticles in an in vitro cell-free tissue

**Fig. 4:**
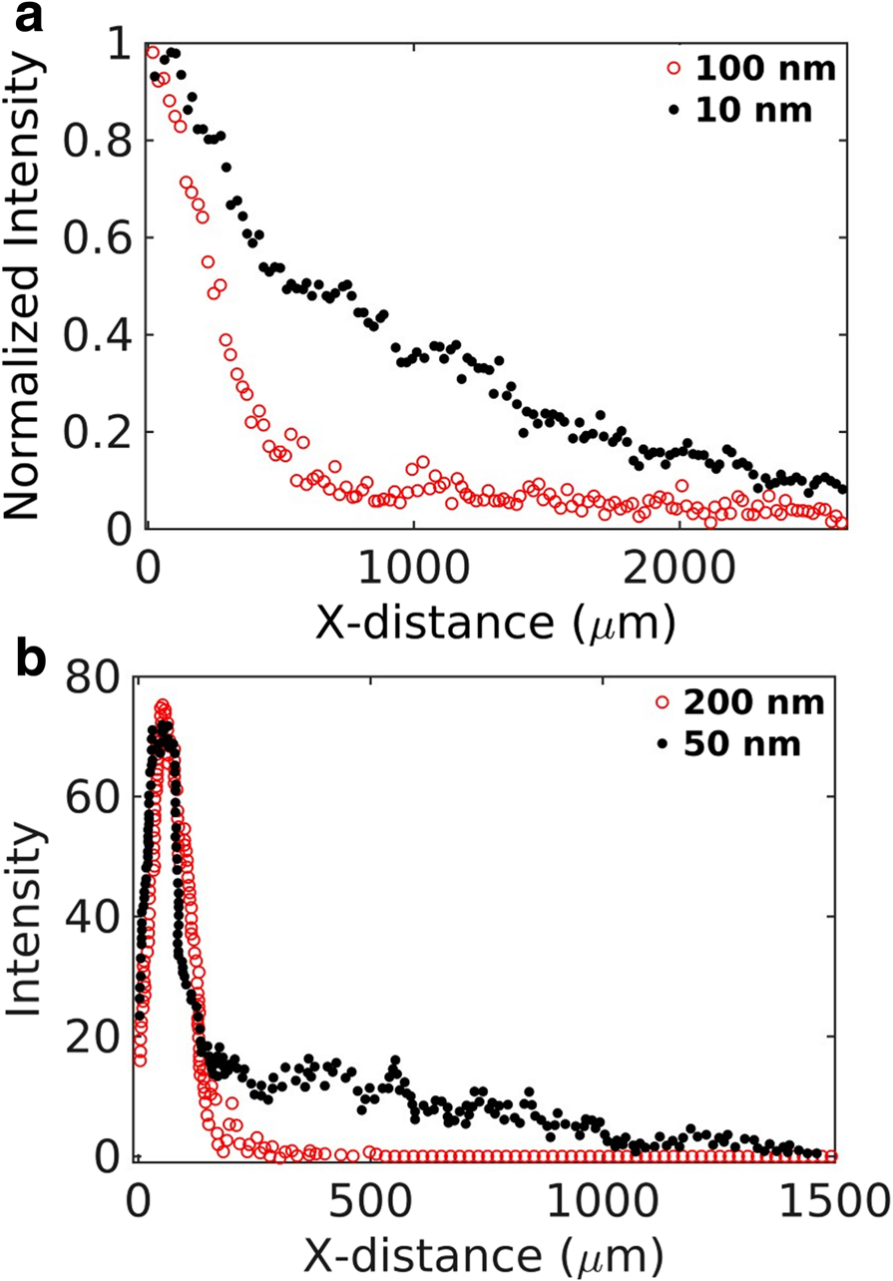
Experimental data adapted from two earlier works [6, 7]. **a** Data from Figure 3H of Wong et al. [6]. The figure compares distribution of 100 nm (red) and 10 nm (black) nanoparticles in collagen in an in vitro experiment. **b** Experimental data adapted from Fig. 5d of Tang et al. [7]. The figure compares tumor tissue distribution of 200 nm (red) and 50 nm (black) particles in an in vivo experiment

In drug-delivery experiments, it is a common practice to employ cell-free tissue systems as a substitute of an in vivo physiological tissue. We first investigated particle size effects on the distribution and penetration of nanoparticles in such in vitro tissue systems. As mentioned previously, the experimental work of Wong et al. [6] studied the effects of particle size in a cell-free collagen matrix (Fig. 4 a). In contrast, Tang et al. [7] investigated particle size effects in in vivo tumor tissues (Fig. 4 b). The collagen matrix used in Wong et al. [6] was devoid of cells and advective transport. An experiment in the study compared the tissue distribution and penetration efficacy of 10 and 100 nm particles. Both particle sizes displayed a broad dispersion across the tissue system. However, the smaller particles revealed a significantly deeper penetration (Fig. 4 a).

**Fig. 5:**
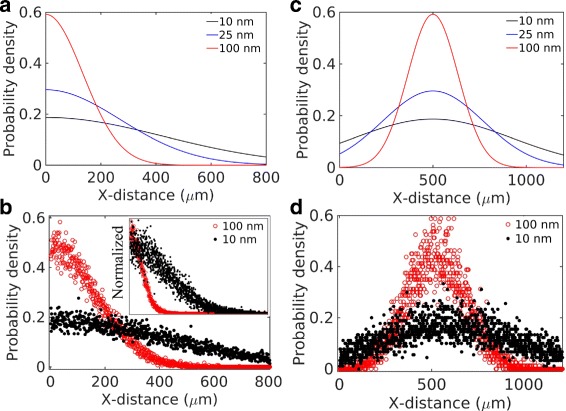
Particle size effects in a cell-free system. **a** Theoretical model (Eq. 6) and **b** simulation considering pure diffusion. **c** Theoretical model (Eq. 7) and **d** simulation considering a small advection (0.05 *μ*m/s) and diffusion

The experimental observation of Wong et al. [6] can be explained with a simple theoretical model. Comparing the tissue domain with a semi-infinite plane in one dimension, the solution of the following equation describes the time-dependent concentration profile (probability density function) of a single particle in the domain:

6 $$ \frac{\partial{G}}{\partial t} = D\frac{\partial^{2} G}{\partial x^{2}} + \delta(x)\delta(t),  $$

where the source term (product of the Dirac delta functions) represents the initial particle location at the origin. *D* is the size-dependent diffusion coefficient (Eq. 5). The solution of this equation is $G(x,t) = (1/\sqrt {\pi D t})exp(-x^{2}/4Dt)$. The solution is similar to a Gaussian distribution in an infinite domain with the exception that the peak height is $1/\sqrt {\pi D t}$ instead of the corresponding Gaussian peak $1/\sqrt {4\pi D t}$, and the solution is valid only in the right half plane (*x*≥0). Figure 5 a represents this analytical solution for three different particle sizes. The diffusion coefficient of each particle size was calculated based on the Einstein-Stokes formula (Eq. 5) and the physical properties of the interstitial fluid listed in Table 1. Figure 5 b shows corresponding results from our simulation for two different particle sizes (10 and 100 nm). The inset of Fig. 5 b shows the normalized curves for a direct comparison with the fluorescence data in [6] (Fig. 4 a).

In a biological tissue, however, it is unlikely to have a purely diffusive motion of particles. In the presence of a small flow (advection) to the right, particle distribution can be described by the following equation:

7 $$ \frac{\partial{G}}{\partial t} = D\frac{\partial^{2} G}{\partial x^{2}} - v\frac{\partial G}{\partial t} + \delta(x)\delta(t),  $$

where *v* is a constant velocity in the X-direction. The solution of this equation, $G(x,t) = (1/\sqrt {\pi D t})exp(-(x-vt)^{2}/4Dt)$, is shown in Fig. 5 c for *v*=0.05 *μ*m/s. Corresponding simulation result is shown in Fig. 5 d. The distribution peaks are shifted by a distance *vt*, as expected. Based on this result, in a cell-free system, it may take only few hundred seconds for a particle to travel tissue-scale distances (few hundred microns). Contrary to this, the in vivo distribution in Tang et al. [7] (Fig. 4 b) clearly indicates that particles travel at a much slower pace in a physiological tissue condition perhaps because of the transport barriers imposed by the cells.

### Size effects of particles in in vivo tissue conditions

We next investigated how particle size may impact the tissue distribution and penetration efficacy in a physiological tissue condition. We were interested in the in vivo tumor tissue distributions reported in Tang et al. [7] (Fig. 4 b). This in vivo data indicated a modestly deeper penetration by the smaller particle but the tissue distribution profiles of the particles were significantly different from those observed in the cell-free collagen sample in [6] (Fig. 4 a). Both particles revealed narrow and overlapping peaks, suggesting a relatively poor tissue dispersion and penetration compared to the cell-free system.

**Fig. 6:**
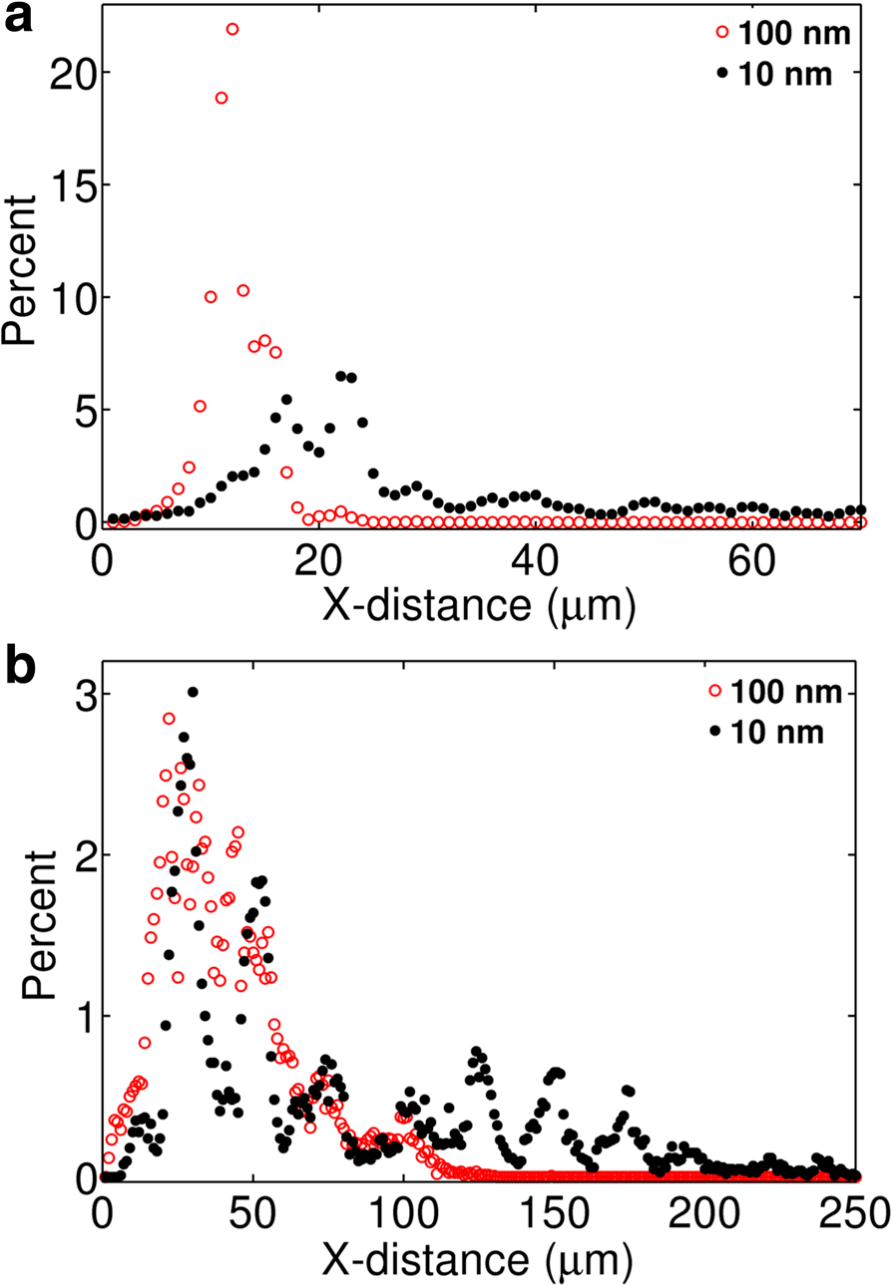
Predicted particle size effects in the presence of cells. The panels represent the following conditions: **a** pure diffusion and cells; **b** advection, diffusion, and cells. All simulations were carried out considering *ρ*=0.01. The fluid velocity at the tissue entry (left edge) was assumed *v*=1 *μ*m/s [9]

We investigated two different scenarios in the presence of cells. In one case, we included only cells and diffusion but no advection (Fig. 6 a). In the other case, we included cells, diffusion, and advection (Fig. 6 b). This latter condition could be a more practical representation of a biological tissue.

Comparing Fig. 6 with Fig. 5, the presence of cells in the model had a dramatic effect on the penetration depth. The dispersion of both the 100 and 10 nm particles were significantly reduced under pure diffusion (Fig. 6 a) as well as under advection and diffusion (Fig. 6 b). The predicted distributions in Fig. 6 b are qualitatively consistent with the experimental observations of Tang et al. [7]. Consistent with the experimental data, the model shows that the peaks of the 10 and 100 nm particle distributions align at the same location though the smaller particle distribution shows a tail stretched further to the right.

Comparing Fig. 5 with Fig. 6, a cell-free in vitro system may provide inaccurate information as to how the particle size affects the distribution and penetration of nanoparticles in biological tissues. Figure 5 indicates the 10 nm particles are significantly more efficient in tissue dispersion, consistent with the experiment of Wong et al. [6]. However, Fig. 6 indicates the difference between the 10 and 100 nm particles may be less pronounced in a real tissue system, where particle motions could be hindered by their interaction with the cell boundaries.

Our analysis above indicates that cell-surface adhesion and capture of particles may significantly compromise the particle size effects in in vivo physiological conditions. In a cell-free system, particle size effects could be more significant due to the unrestricted diffusion, which is directly determined by particle size. In contrast, in the presence of cells, diffusion plays a less significant role. Therefore, in vivo interstitial transport behavior of particles could be predominantly determined by the barriers imposed by the cell boundaries.

**Fig. 7:**
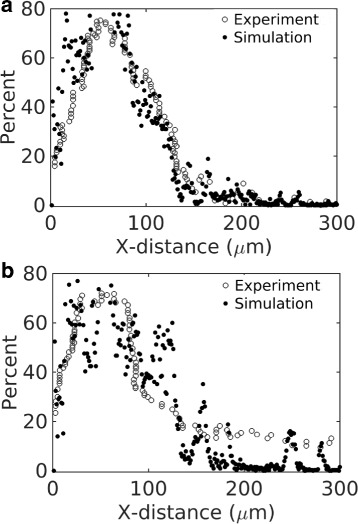
Comparison between simulation and experiement. The open circles represent the experimental data of Fig. 4 b (plotted in a different scale). The filled circles represent simulation. **a** Particle size is 200 nm. **b** Particle size is 50 nm

We next used the model to capture the experimental data of Tang et al. [7] (Fig. 4 b). A direct fit between the model and the data was not possible due to the missing information on the exact experimental time frame and tissue properties, which include cell density and interstitial fluid properties, fluid velocity, and particle capture rate by cells. We simulated the system for 10,000 seconds and attempted to match the position of the distribution peaks for the two particle sizes reported in [7]. The match between the simulation and data (Fig. 7) required variations in the inlet fluid velocity (*v*_*in*_) and the probability of particle capture per collision (*ρ*), leading to *v*_*in*_=4 *μ*m/s and *ρ*=0.001.

The small value of *ρ* indicates that a particle gets captured after many contacts (collisions) with the cell boundary. At this range of *ρ*, we found that the particle distribution profiles were less sensitive to the value of *ρ* in our simulations. The distributions were primarily determined by the fluid velocity and duration of the simulation. It should be noted that the parameter *ρ* does not capture the possibility of particle dissociation (reversible binding). Replacing this simple probabilistic construct based on *ρ* with more mechanistic details of particle uptake [26] and complementary quantitative experiments might shed light on particle uptake rate by cells in biological tissues.

### Effects of cellular uptake rate on tissue dispersion and penetration

**Fig. 8:**
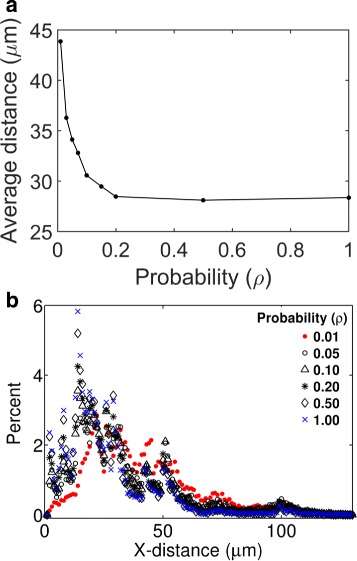
Predicted effects of cellular uptake rates on tissue distribution of nanoparticles. **a** Mean depth of tissue penetration by particles as a function of *ρ*. The mean depth of penetration represents the average of the horizontal positions (X-coordinate) of 16,000 simulated particles in 10^4^ seconds after their tissue entry. **b** Histograms showing distribution of the nanoparticles. Each histogram corresponds to a different value of *ρ*, as indicated in the figure legend

Our previous analysis led us to further investigate how cells influence the tissue distribution of particles. In Fig. 8, we investigated the effects of *ρ* on the tissue penetration efficacy of 100 nm nanoparticles. Figure 8 a shows the mean depth of penetration as a direct function *ρ*. Figure 8 b shows the tissue distribution profiles at different values of this parameter. As seen in the figures, the penetration depth and the distributions were insensitive in the range 0.1<*ρ*<1. However, there was a noticeable change in the penetration depth and distributions in the range *ρ*<0.1.

**Fig. 9:**
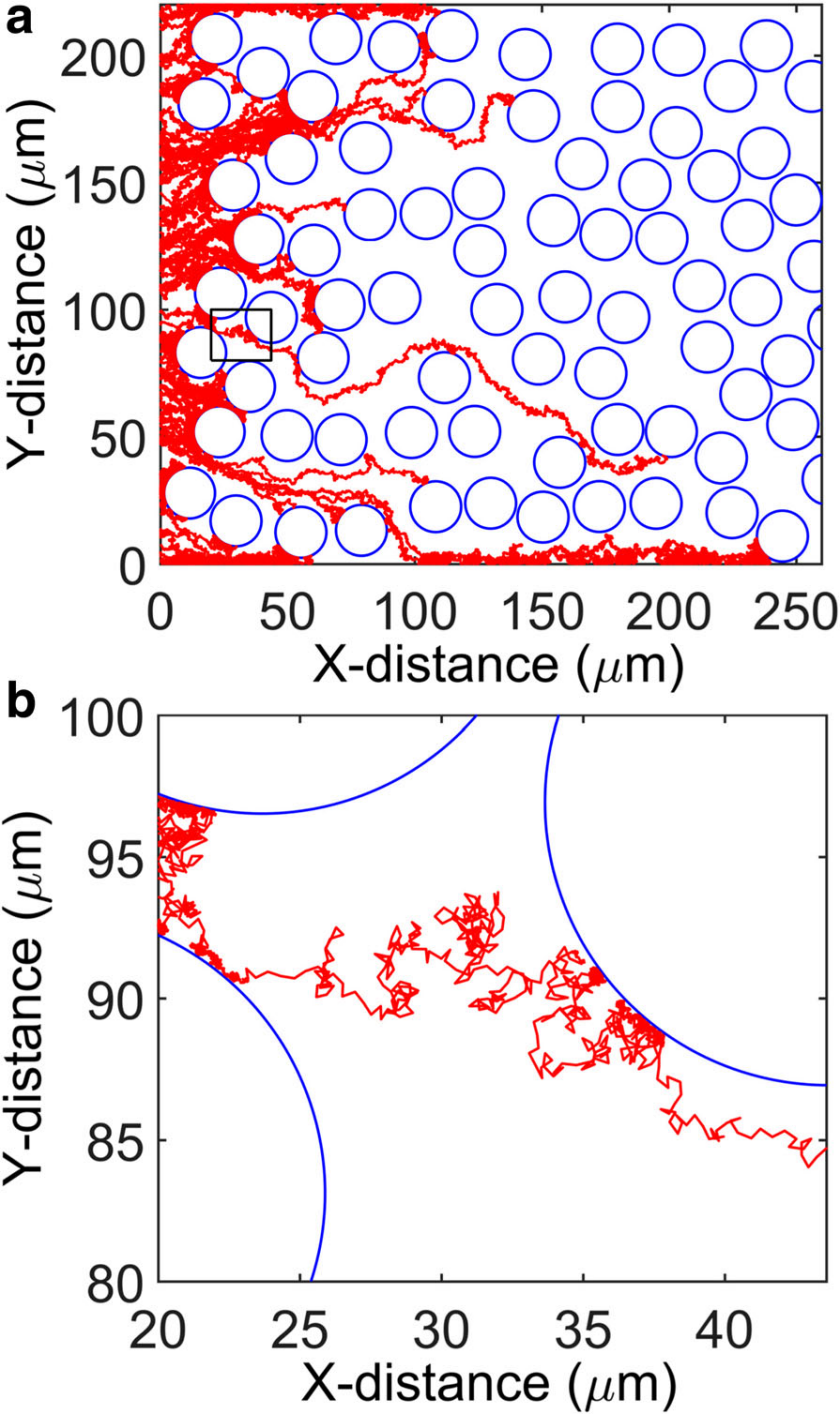
Representative travel paths of simulated nanoparticles. **a** Travel paths of 100 nanoparticles in the tissue domain. The particles are of identical size (100 nm radius). **b** A zoomed-in view showing a single particle travel path and its interaction with a cell boundary. Panel B corresponds to the small region in Panel A marked by a rectangle

The results above indicate a non-linear relationship between the cellular uptake rate and tissue penetration depth. This nonlinearity could reflect the fact that the overall rate of cellular uptake is determined not only by *ρ* but also by the mean number of collisions a particle makes with the cell boundaries. If a particle on average makes *C* number of collisions with any cell boundary, the probability that it will get captured is *ρ* *C*. As for example, with *ρ*=0.1 and *C*≥10, particle can get captured with probability 1 upon its encounter with a cell. Therefore, a further increase in *ρ* beyond 0.1 could have little impact on the overall capture rate. Our adaptive algorithm takes fine-resolution time step (*δ* *t*) near the solid (cell) boundaries, as discussed in Materials and Methods. As illustrated in Fig. 9 (also in Additional file 2: A and B), the fine resolution *δ* *t*=10^−3^ second near the cell boundaries allows a particle to make many collisions with a cell before it gets captured. Therefore, the actual rate of cellular uptake could be high even though *ρ* is small. In our simulations, the default value of *ρ* is 0.01 (Table 1).

### Model prediction sensitivity to time steps

**Fig. 10:**
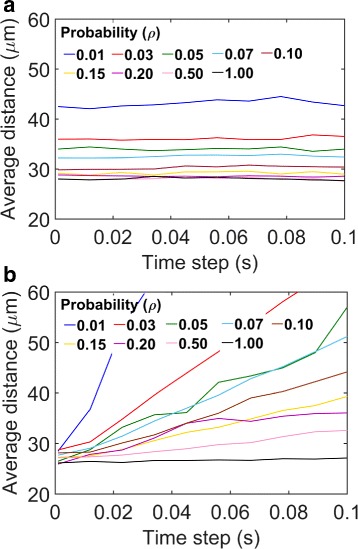
Effect of time step *Δ* *t*_*m*_ variation on model predictions. **a** Average tissue penetration by particles as a function of *Δ* *t*_*m*_. Each curve corresponds to a different value of *ρ* (the probability of particle capture by a cell in a collision between the particle and the cell.) **b** The analysis of Panel A is repeated using a non-adaptive BD algorithm based on Rejniak et al. [9]

Because our adaptive algorithm selects time steps over a wide range (*Δ* *t*_*m*_=0.1<*Δ* *t*<*δ* *t*=10^−3^ s), we wanted to investigate if the predictions in Fig. 10 could be sensitive to the selection of time steps. Therefore, we varied the upper bound (*Δ* *t*_*m*_) in the range 10^−3^−0.1 s to enforce different resolution of time steps in the simulation algorithm. For each *Δ* *t*_*m*_, we simulated 16,000 nanoparticles for 10^4^ s and then calculated the mean depth of tissue penetration by these particles. We carried out this analysis for different values of *ρ*. Corresponding plots are provided in Fig. 10 a. As seen in the figure, the predictions remained insensitive to the *Δ* *t*_*m*_. This robustness reflects the fact that the algorithm adapts to smaller steps when particles are in close proximity to the cell boundaries regardless of the value of *Δ* *t*_*m*_.

However, it is important to note that *Δ* *t*_*m*_ cannot be assigned an arbitrarily large value. A smaller *Δ* *t*_*m*_ is needed to approximate particle velocities to the local fluid velocity. A large *Δ* *t*_*m*_ enables the particles to advance with large steps. As a result, local velocity fields before and after the jump could be significantly different, thus introducing larger inaccuracies in the velocity approximation for the particles.

In Fig. 10 b, we performed the same analysis using a non-adaptive algorithm, where we kept the time step size constant. This fixed time-step algorithm is similar to the algorithm of Rejniak et al. [9]. Contrary to our approach, the algorithm of Rejniak et al. [9], however, treated particles (drug molecules) as point objects. The algorithm moved the particles based on a fixed time step and rejected the moves in case of a conflict with the cell positions. The algorithm also assumed an interaction layer of 0.25 *μ*m around each cell periphery. A particle was considered captured by a cell immediately upon its arrival within the 0.25 *μ*m interaction layer. We took into account these features of the Rejniak model with the following exceptions: 1) Instead of treating the particles as points, we treated them as circular objects of 100 nm radius, as in our model; and 2) Instead of assuming an immediate particle capture within the interaction layer, we incorporated a capture probability 0≤*ρ*≤1 in the layer. The predictions made by this algorithm at different selections of the time step size and *ρ* are shown in Fig. 10 b. Clearly, the predictions were sensitive to the choice of the step size. This sensitivity is expected because the rate of particle capture by cells in this algorithm should depend on the thickness of the interaction layer and the relative choice of the time step size. For a thinner interaction layer, a particle would be less likely to hit the layer if advanced based on a fixed step. Similarly, an increase in the time step size would also reduce the possibility of hitting the interaction layer. Thus, the fixed time step algorithm should underestimate the rate of particle capture (cellular uptake) and overestimate the tissue penetration depth if a smaller interaction layer or larger time step is chosen. Moreover, due to the fixed (and large) time step size in the algorithm of Rejniak et al. [9], many particle moves might be rejected due to the conflicts with the cell positions.

As mentioned before, *δ* *t*=10^−3^ represents the smallest time step in our model. Because of the no-slip boundary condition, particle motion near a cell boundary is primarily driven by diffusion. Thus, the length of a particle jump near a cell boundary can be estimated based on pure diffusion: $|\boldsymbol {S}| \approx |\vec {\boldsymbol {S}}_{d}| = \sqrt {4D\delta t}$. For the fluid properties and temperature listed in Table 1, this jump size becomes comparable to the size of the particle. Therefore, it is a sufficiently small step size to capture the fine resolution details of interactions occurring at the particle-cell interface.

## Discussion

In this work, we developed a multiscale Brownian Dynamics algorithm to study particle transport behavior in biological tissues. using the approach, we investigated particle size effects on tissue distribution and penetration reported in two experimental studies. Our analysis focused on how these behaviors may vary in cell-free artificial tissue systems and in vivo tissue conditions.

Our multiscale algorithm can be generally applicable to modeling advection-diffusion systems involving heterogeneous porous media. The approach we have implemented is inspired by two previous modeling works [9, 27]. Earlier, Monine et al. [27] developed a time-adaptive Brownian Dynamics (BD) algorithm to study enzyme-substrate reaction in the plasma membrane of cells. Recently, Rejniak et al. [9] used the Method of Regularized Stokeslets (MRS) [8] to study drug molecule transport in biological tissues. Both these models treated the mobile particles (substrate and drug molecules, respectively) as point particles while considering their stationary reaction or binding partners (enzyme molecules and cells, respectively) as circular objects. In our model, we combined the time-adaptive feature of the Monine model with the MRS. This combination enabled multiscale modeling of particle transport under both advection and diffusion while capturing high-resolution details of particle interaction with the cell boundaries. Contrary to the point particle assumption in the Monine model and Rejniak model, we considered the mobile nanoparticles as spherical objects occupying space in the two-dimensional membrane.

Contrary to the general perception, our study revealed less significant effects of particle size on their intra-tissue distribution and penetration. Our analysis shows that in vitro tissue systems, being devoid of cells and convective flow, may result in misleading conclusions regarding the transport behavior of particles in the biological tissues. Here, we limited our focus to particle size only. However, the multiscale algorithm can be extended to incorporate other design attributes of particles, such as geometry and surface ligands. This extension will allow mechanistic interrogation of how these parameters affect the transport behavior of particles in biological tissues.

In the model, we treated the nanoparticles as mutually non-interacting objects. In the model, the particles do not collide or form aggregates. This consideration is based on the assumption that physiological tissue concentrations of drug-delivery nanoparticles are small. Apparently, there is no report on the mutual interactions of drug-delivery nanoparticles in the physiological tissue conditions. It has been reported that 1% of intravenously injected particles can reach the target tissue [28, 29]. Therefore, from the injection of 1 ml solution containing 100 million particles/ml [30], only a 1 million particles are expected to reach the target tissues. Thus, for 100 nm radius particles, the estimated volume fraction of particles in the target tissues could be in the order of 10^−9^ assuming 1 cm^3^ of tumor tissue volume (a single tumor or many smaller tumors). At this volume fraction, their non-specific collision is unlikely or less important considering many other cellular proteins and biomolecules that could present at comparable amounts.

Our model does not consider the effects arising from the surface charges of particles or van der Waals forces acting between a particle and a cell. Moreover, in a body fluid, soluble biomolecules may interact with nanoparticles and form a coating or biocorona over the particle surface [18–21]. Formation of biocorona modifies the surface properties of particles. At present, the quantitative aspects of biocorona formation and how it modifies the particle surface properties and tissue interaction are not well-understood. Therefore, rather than explicitly incorporating these other properties (van der Waals and biocorona effects), we used a phenomenological parameter *ρ* in the model that accounts for a lumped measure of the affinity of interaction between a nanoparticle and a cell. Nevertheless, for a quantitative understanding of these other phenomena influencing tissue interactions of particles, it is crucial to explicitly address them in a mechanistic model. The Brownian Dynamics-based framework presented here could serve as an initial platform towards this direction. The framework could be extended to capture these other types of particle- and tissue-specific physicochemical parameters. Integration of such predictive mechanistic models with complimentary experiments could be essential for a quantitative elucidation of these other effects on drug delivery nanoparticles in biological tissues [31].

We considered nanoparticle velocity to be the same as the local fluid velocity while ignoring the influence of the particles on the velocity field. It is possible that large particles also modify the local velocity fields at the micro scale. However, nanoparticles are of the same dimension as many cellular proteins, biomolecules, and solute particle. Our model is based on existing models where nanoparticles velocities were considered to be the same as fluid velocities in the porous media [32–35]

Our modeling approach may be expanded for spatiotemporal modeling biochemical network systems. The rule-based modeling (RBM) approach [36–38] provides unique capability to model biochemical network systems by taking into account the coarse-grained structural details of protein molecules [39, 40]. However, most of the early RBM tools were developed aiming at non-spatial modeling. Recently, the RBM tools Kappa [41], Simmune [42], and BioNetGen [43] are being added with new capabilities for spatiotemporal modeling. The molecular dynamics (MD) simulation is used to model protein structures with atomistic details [44]. But MD can deal with very short time scales, and not scalable for biochemical network modeling considering a large number of species and their structural details.

## Conclusions

We have developed and applied a robust multiscale simulation method for mechanistic modeling of particle transport in porous media. By combining a new time-adaptive BD simulation algorithm with the Method of Regularized Stokeslets (MRS), our method provides a unique capability to model particle transport considering particle size and particle-cell interactions in a heterogeneous biological tissue. Using the approach, we have investigated particle size effects on their distribution and penetration in biological tissues. Contrary to the general perception, we show that particle size may play a less significant role in particle transport in the physiological tissue conditions. We show that, in the presence of cells, the effects arising from particle size difference is small. Particle penetration and distribution is primarily determined by particle-cell interactions. Our study underscores the roles of advective transport and cells that are often ignored in artificial tissue systems of in vitro experiments.

## Additional files

Additional file 1: Model Source Code. The compressed folder, S1_File.zip, contains necessary files and instructions to run a simulation. The file named main.cpp contains the C++ source code. The file named README.txt contains necessary instructions to compile the code and execute the simulation. (ZIP 8 kb)

Additional file 2: Time Adaptive Motion of a Particle. (A) Movie file S1_Video_A.mp4 shows the time-adaptive motion of a single nanoparticle in the interstitial space and near the cell boundaries. Only the motion of the particle center is shown. (B) Movie file S1_Video_B.mp4 shows a more zoomed-in view. Both the particle and the cell are represented by circles. The circles are scaled based on their relative size in the model (particle radius 100 nm and cell radius 10 *μ*m). (ZIP 821 kb)
